# Three allele combinations associated with Multiple Sclerosis

**DOI:** 10.1186/1471-2350-7-63

**Published:** 2006-07-26

**Authors:** Olga O Favorova, Alexander V Favorov, Alexey N Boiko, Timofey V Andreewski, Marina A Sudomoina, Alexey D Alekseenkov, Olga G Kulakova, Eugenyi I Gusev, Giovanni Parmigiani, Michael F Ochs

**Affiliations:** 1 Department of Molecular Biology and Medical Biotechnology, Russian State Medical University, 15 3d Cherepkovskaya ul., Moscow 121552, Russia; 2 Cardiology Research Center, 15 3d Cherepkovskaya ul., Moscow 121552, Russia; 3 Bioinformatics Laboratory, GosNIIGenetika, 1 1st Dorozhny pr., Moscow 117545, Russia; 4 Department of Neurology and Neurosurgery, Russian State Medical University, 1 Ostrovitianova ul., Moscow 117997, Russia; 5 Departments of Oncology, Pathology and Biostatistics, Johns Hopkins University, 550 North Broadway, s. 1103, Baltimore, Maryland 21205, USA; 6 Fox Chase Cancer Center, 333 Cottman Avenue, Philadelphia, Pennsylvania 19111, USA

## Abstract

**Background:**

Multiple sclerosis (MS) is an immune-mediated disease of polygenic etiology. Dissection of its genetic background is a complex problem, because of the combinatorial possibilities of gene-gene interactions. As genotyping methods improve throughput, approaches that can explore multigene interactions appropriately should lead to improved understanding of MS.

**Methods:**

286 unrelated patients with definite MS and 362 unrelated healthy controls of Russian descent were genotyped at polymorphic loci (including SNPs, repeat polymorphisms, and an insertion/deletion) of the *DRB1*, *TNF*, *LT*, *TGFβ1*, *CCR5 *and *CTLA4 *genes and TNFa and TNFb microsatellites. Each allele carriership in patients and controls was compared by Fisher's exact test, and disease-associated combinations of alleles in the data set were sought using a Bayesian Markov chain Monte Carlo-based method recently developed by our group.

**Results:**

We identified two previously unknown MS-associated tri-allelic combinations:

-509TGFβ1*C, DRB1*18(3), CTLA4*G and -238TNF*B1,-308TNF*A2, CTLA4*G, which perfectly separate MS cases from controls, at least in the present sample. The previously described DRB1*15(2) allele, the microsatellite TNFa9 allele and the biallelic combination *CCR5*Δ32, DRB1*04 were also reidentified as MS-associated.

**Conclusion:**

These results represent an independent validation of MS association with DRB1*15(2) and TNFa9 in Russians and are the first to find the interplay of three loci in conferring susceptibility to MS. They demonstrate the efficacy of our approach for the identification of complex-disease-associated combinations of alleles.

## Background

Investigation of polygenic human diseases, which arise from the combined contribution of multiple independently acting and/or interacting polymorphic genes, remains a great challenge [1-3]. A common constituent of the complexity that underlies the susceptibility to polygenic diseases is heterogeneity [3,4]. MS [MIM 126200] is an immune-mediated hereditary disease [5,6], and can be considered as a prototype for polygenic human diseases [4]. The results of linkage-based whole genome screen studies [7,8] and a global meta-analysis [9,10] document the concept that MS is the result of the interaction of several genes. The effects of individual genes are small or modest, making association studies more informative than others because of their greater statistical power [1,11]. Association testing is extensively employed in candidate-gene studies, which are usually conducted in population-based case-control studies. To date, the HLA class II DRB1*1501/DQA1*0102/DQB1*0602 (DR2) haplotype is the only region repeatedly confirmed as being associated with MS in most Caucasians [5,10]. Other candidate genes for MS predisposition studies have been selected mainly because their encoded proteins are involved in autoimmune pathogenesis. These include genes for immunorelevant molecules such as cytokines, cytokine receptors, immunoglobulins, T-cell receptors and specific adapter protein, potential autoantigens of the myelin sheath, ICAM1, and others [12].

Some studies searched for candidate gene combinations as MS risk factors; however, these studies have not yet extended beyond MS associations with alleles of each candidate gene coupled with HLA *DRB1 *alleles [13-19]. To examine the possibility that the combined effect of definite genes is a risk factor for a polygenic disease, it is necessary to explore a massive number of potential combinations of allelic variants identified at candidate-gene polymorphic loci. This number grows exponentially with the number of candidate variants that may interact, causing computational and statistical restrictions on the use of standard enumerational methodologies. In a recent paper [20], we described a novel algorithm based on Markov chain Monte Carlo exploration using a Bayesian statistical basis, APSampler, which allows the exploration of genotypes tied to phenotypic trait levels to identify possible combinations of allelic variants at multiple loci that could affect disease development. The aim of this study is to simultaneously examine multiple candidate genes in single groups of unrelated MS patients and healthy unrelated controls, all of Russian descent, and to search for disease-associated combinations of allelic variants at multiple loci using our novel nonparametric methodology [20].

## Methods

### Subjects and DNA samples

Two hundred eighty six unrelated patients (110 men and 176 women, mean age 33 ± 12 years) had a diagnosis of MS [21]. Of these 187 had a relapsing-remitting MS course, 39 a primary progressive MS course, and 60 were secondary progressive. The mean age at onset was 23 ± 9 years. Three hundred sixty-two unrelated controls (203 men and 159 women, mean age 30 ± 11 years) were studied. All controls were free of acute or chronic internal and neurological diseases as determined by physical examinations. All subjects were living in the Moscow area; both their parents were ethnic Russians. Informed consent to the study was obtained from all participants and was approved by the local Ethical Committee. Blood was adjusted to 25 mM EDTA. Genomic DNA was isolated from 5 mL of peripheral blood by phenol-chloroform extraction using standard procedures.

### Genotyping

Investigated polymorphic loci (including SNPs, repeat polymorphisms, and one insertion/deletion) and the numbers of genotyped MS patients and controls for each marker are presented in Table 1.

**Table 1:** Polymorphous loci at or near genes of immune response included in the database

Gene/marker	Chromosome localization	Polymorphism type* [refSNP ID]**	Names of alleles considered	Method of analysis (the restriction endonuclease used)	Numbers of genotyped MS patients/controls
*DRB1*	6p21	Allele groups corresponding to serological specificities DR1-DR18(3)	01–18(3) [44]	PCR-SSP	229/314
Microsatellite TNFa	6p21	(AC)_n_	a1-a13[23]	Nested PCR	121/103
Microsatellite TNFb	6p21	(TC)_n_	b1-b7[23]	Nested PCR	120/96
		SNP -376A→G [rs1800750]	A, G	PCR-SSP	202/146
*TNF*	6p21	SNP -308G→A [rs1800629]	A1, A2	PCR-SSP	223/222
		SNP -238A→G [rs361525]	B1, B2	PCR-RFLP (*Bam*HI)	165/112
*LT*	6p21	SNP +252G→A	N1, N2	PCR-RFLP (*Nco*I)	205/150
		SNP +319C→G	H1, H2	PCR-RFLP (*Asp*HI)	202/147
		SNP -509C→T [rs17551290]	C, T	PCR-SSO	119/295
		SNP +72 wild type→C insertion	wt, ins		198/340
*TGFβ1*	19q13	SNP +869T→C (10Leu→Pro)	T, C		150/248
		SNP +915G→C (25Arg→Pro)	G, C		157/248
		SNP +1632C→T (263Thr→Ile)	C, T		178/109
*CCR5*	3p21	Wild type→32 base pair deletion	wt, Δ 32	PCR	221/355
*CTLA4*	2q33	SNP +49A→G (17Thr→Ala) [rs231775]	A, G	PCR-RFLP (*Bst*EII)	168/209

Abbreviations used: *CCR5 *– CC chemokine receptor 5 gene; *CTLA4 *– cytotoxic T-lymphocyte-associated protein 4 gene; *DRB1 *– major histocompatibility complex class II DR β-chain gene 1; *LT *– lymphotoxin α gene; *TGFβ1 *– transforming growth factor β-1 gene; *TNF *– tumor necrosis factor gene; PCR – polymerase chain reaction; RFLP – restriction fragment length polymorphism; SSO – sequence-specific oligonucleotides; SSP – sequence-specific primers; ins – insertion; wt – wild type.

* All positions of the SNPs are indicated relative to transcriptional start sites except *CTLA4 *SNP +49, where +49 is the position from translational start site.

** RefSNP IDs (rs#) are given for SNPs submitted in PubMed NCBI dbSNP.

#### HLA DRB1 gene

For genomic typing of the *DRB1 *gene, a sequence-specific primer (PCR-SSP) technique was used. The two-step PCR allowed amplification of all known *DRB1 *alleles and their separation into groups corresponding to the specificities from DR1 to DR18 [22].

#### TNFa and TNFb microsatellites

For a length polymorphism analysis of (AC)_n _and (TC)_n _microsatellites, designated as TNFa and TNFb, which are located 3.5 kb upstream of the *LT *gene, nested PCR was used. The second PCR was carried out in the presence of [α-^32^P]dATP, then PCR products were treated by the Klenow fragment of DNA polymerase I and electrophoresed in an 8% polyacrylamide sequencing gel [23].

#### SNPs in TNF gene

Genotyping of the -238A→G polymorphism was performed by analysis of restriction fragment length polymorphism of PCR products (PCR-RFLP method). PCR amplification was carried out using the exactly homologous forward primer, while the reverse primer contained two sequence mismatches, which made it possible to evaluate mutation status using the restriction enzyme *Bam*HI [24]. Analysis of the -308G→A polymorphism was performed by PCR-SSP [25]. For genotyping of the -376A→G polymorphism, the PCR-SSP method was also used. The forward primers are 5'-CTT TTT CCT GCA TCC TGT CTG GAA *A*-3' for -376A and 5'-CTT TTT CCT GCA TCC TGT CTG GAA *G*-3' for -376G, the common reverse primer was 5'-TTC TGT CTC GGT TTC TTC TCC ATC G-3'. These primers were constructed using free on-line software GeneFisher-Interactive PCR Primer Design [26]. PCR was performed in 10 μL volumes containing 0.1 units of Taq polymerase (from SileksM, Moscow, Russia), 200 ng of genomic DNA, 5 pmol of the reverse PCR primer, 5 pmol of the sequence-specific PCR primer for allele A or 2.5 pmol of the sequence-specific PCR primer for allele G, four dNTPs (each at 5 mM), 70 mM Tris HCl pH 9.0, 20 mM (NH_4_)_2_SO_4_, 0.025% Tween 20, 0.025% NP-40, 1.0 mM MgCl_2_. Thermocycling consisted of 35 cycles of 92°C for 60 s, 60°C for 90 s, 72°C for 90 s. The resulting fragment length was 238 bp.

#### SNPs in LT gene

Genotyping of the +252G→A and +319C→G polymorphisms was performed by PCR-RFLP method. For analysis of the SNP +252G→A, PCR amplification was performed and the product aliquots were digested using the restriction enzyme *Nco*I[27]. In parallel, other aliquots of the same PCR products were digested using the restriction enzyme *Alw*21I (*Asp*HI) for analysis of SNP +319C→G [24].

#### SNPs in TGFβ1 gene

For genotyping of five SNPs in the *TGFβ1 *gene, four PCR fragments were obtained (one PCR fragment was common for the SNP +869T→C (10Leu→Pro) and SNP +915G→C (25Arg→Pro)). Then resulting fragments were immobilized on Hybond-N+ membrane (Amersham Pharmacia Biotech) followed by hybridization with sequence-specific oligonucleotide probes (PCR-SSO method). PCR conditions, primers and probes were as those described in [28], hybridization procedure was modified according to [29] as follows. Prehybridization was performed for at least 2 h at 52°C in 3.0 M tetramethylammonium chloride (TMAC), 50 mM Tris HCl pH 8.0, 2 mM EDTA, 5×Denhardt's solution, 0.1% SDS, 100 ng/mL heat-denatured herring sperm DNA and 10–20 pmol/mL of unlabeled oligonucleotide probe specific for the other allele. Then 10–20 pmol/mL of [γ-^32^P]ATP-labeled allele-specific oligonucleotide was added and hybridization was performed for at least 2 h at 52°C. The membranes were washed at room temperature in 2×SSC for 30 min followed by 2 × 15 min in 2×SSC, 0.1% SDS. Then the membranes were washed twice at 58°C in 3.0 M TMAC, 50 mM Tris HCl pH 8.0, 2 mM EDTA, 0.1% SDS, rinsed in 2×SSC and autoradiographed.

#### 32 base pair deletion in CCR5 gene

*CCR5 *genomic typing was performed using one-step PCR with primers flanking the region of the 32-nucleotide deletion [30].

#### SNP in CTLA4 gene

Genotyping of SNP +49A→G in *CTLA4 *gene was performed by PCR-RFLP analysis using the restriction enzyme *Bst*EII [31].

#### Database

The genotypes and personal data for all patients and controls were entered into a database, together with clinical characteristics for patients. Microsoft Visual FoxPro was used to develop a standalone database management system that was used for input and analysis of the data.

### Search algorithm

We used the APSampler algorithm [20] that identifies combinations (patterns) of alleles at different loci that are potentially associated with a phenotypic trait. Here, we provide a brief description of the algorithm. The *a posteriori *probability for support of each pattern given the data is evaluated using a likelihood obtained from a battery of conditional rank sum statistics that insures that each pattern is evaluated after removing the effect of other patterns, as in a statistical adjustment for multiple regression. Each step of the algorithm is an update of a current set of allelic patterns by a variation of the Metropolis-Hastings algorithm [32,33]. The sets of patterns that receive high support from the data are stored as potential results. For each pattern, only those individuals that have all the pattern's alleles genotyped were considered. Thus the algorithm proceeded from incomplete genotypic information, temporarily omitting those individuals that cannot be unambiguously classified into the pattern carriers and non-carriers at a given step. The executable files for Win32 console and for FreeBSD, together with documentation, are available free to academic users (contact AVF or MFO).

In the current application to the MS case-control data, the prior probability that a locus has no effect on the phenotype was set to 0.99. This favored *a priori* the case in which each locus has no effect on phenotype, so that the search would not explore patterns with a very large number of alleles, which are unlikely to be reliably investigated in this data set. We looked for sets of 2 or 3 patterns.

### Statistical analysis

To compare allelic distributions in MS patients and controls and to verify the APSampler's results, we used Fisher's exact test. The analysis was performed using the GraphPad InStat software package and original computer scripts under Microsoft Visual FoxPro. As a statistical control for systematic genotyping error and population stratification, the expected genotype proportions according to the Hardy-Weinberg equilibrium were calculated and compared to observed genotypes. For polymorphic *DRB1 *and TNFa loci, the *p-*values were corrected for the number of comparisons (13 in case of *DRB1 *allele groups and 12 in case of TNFa alleles) according to the Bonferroni method. The *p-*values and corrected *p-*values (*p*_*corr*_) were considered to be significant at a level smaller than 0.01. The odds ratio (OR) was calculated with a 95% confidence interval (CI).

For the haplotype frequency estimation, for the linkage disequilibrium test, and for the Hardy-Weinberg disequilibrium test, the population genetics data-analyzing software Arlequin (version 2.0) [34] was used. We considered a linkage disequilibrium or a Hardy-Weinberg disequilibrium to be significant when the *p*-value from the Arlequin output was less than or equal to 0.05. For estimation of haplotype frequencies, the default parameters were used. To assess the significance of the APSampler-discovered patterns, we computed the Empirical Bayes false discovery rate, which is the estimated fraction of patterns unrelated to the phenotype among those that have a significance score as high or higher than the pattern reported [35]. The vector of disease labels was permuted in a balanced way by assigning the same number of diseased and healthy individuals to two groups generated by permutation. We repeated the permutation 100 times and examined all possible combinations with the same number of alleles as the pattern considered. For each permutation and each combination, we computed a test of association and counted the fraction of these tests that were larger than the observed test for the pattern.

## Results

**Figure 1:**
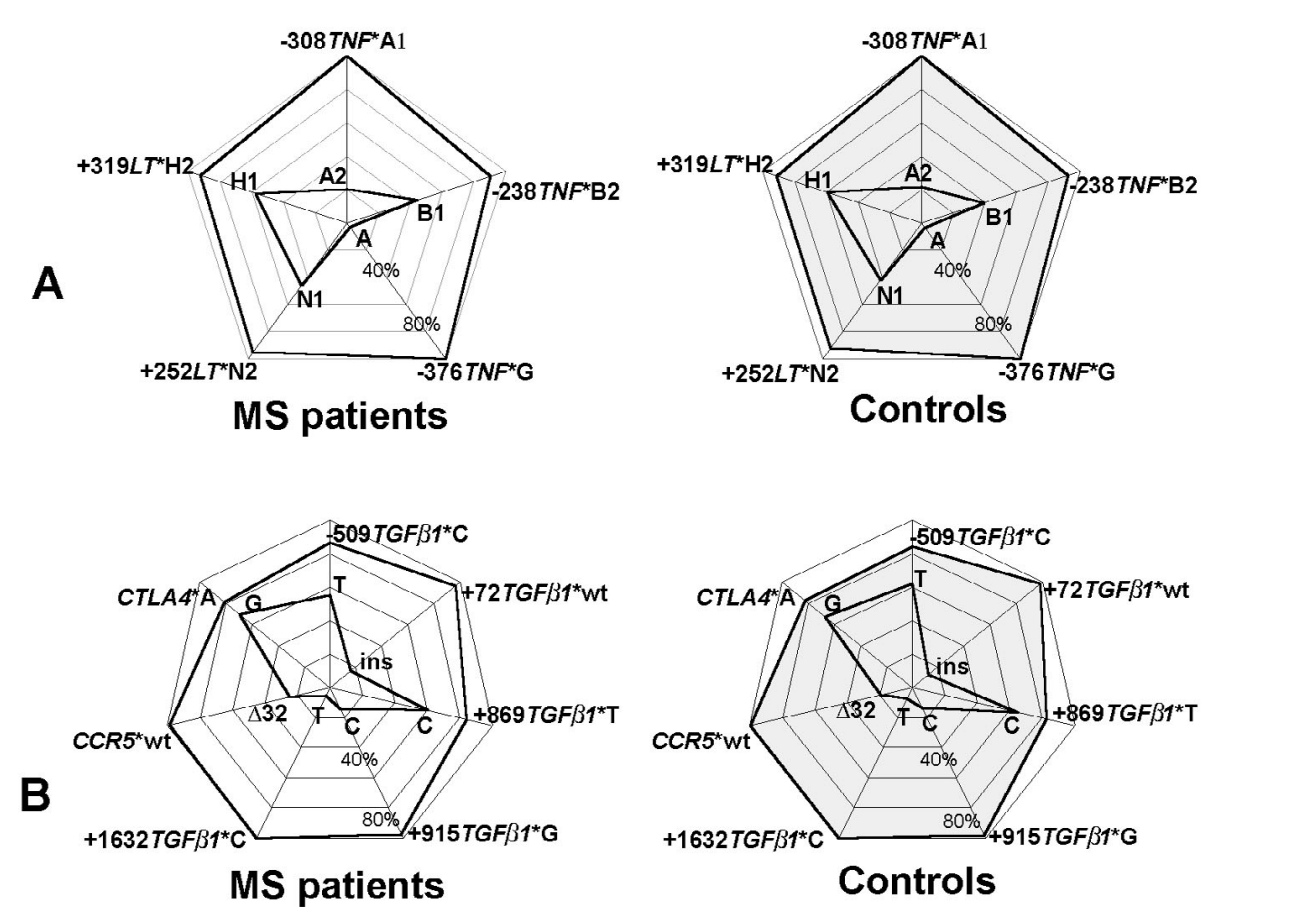
**Phenotypic frequencies (%) of SNP alleles in Russian MS patients and healthy individuals**. The numbers of typed individuals are given in **Table 1**. **A: ***TNF *and *LT *gene polymorphic regions; **B: ***TGF β1*, *CCR5 *and *CTLA4 *gene polymorphic regions. For each SNP, phenotypic frequencies of both alleles are shown on the same axis of a radar chart; thick lines join all common and all rare alleles of SNPs presented, and SNP allele names are indicated on the vertices. The same data are presented in Additional Table 1 [see Additional File 4].

**Figure 2:**
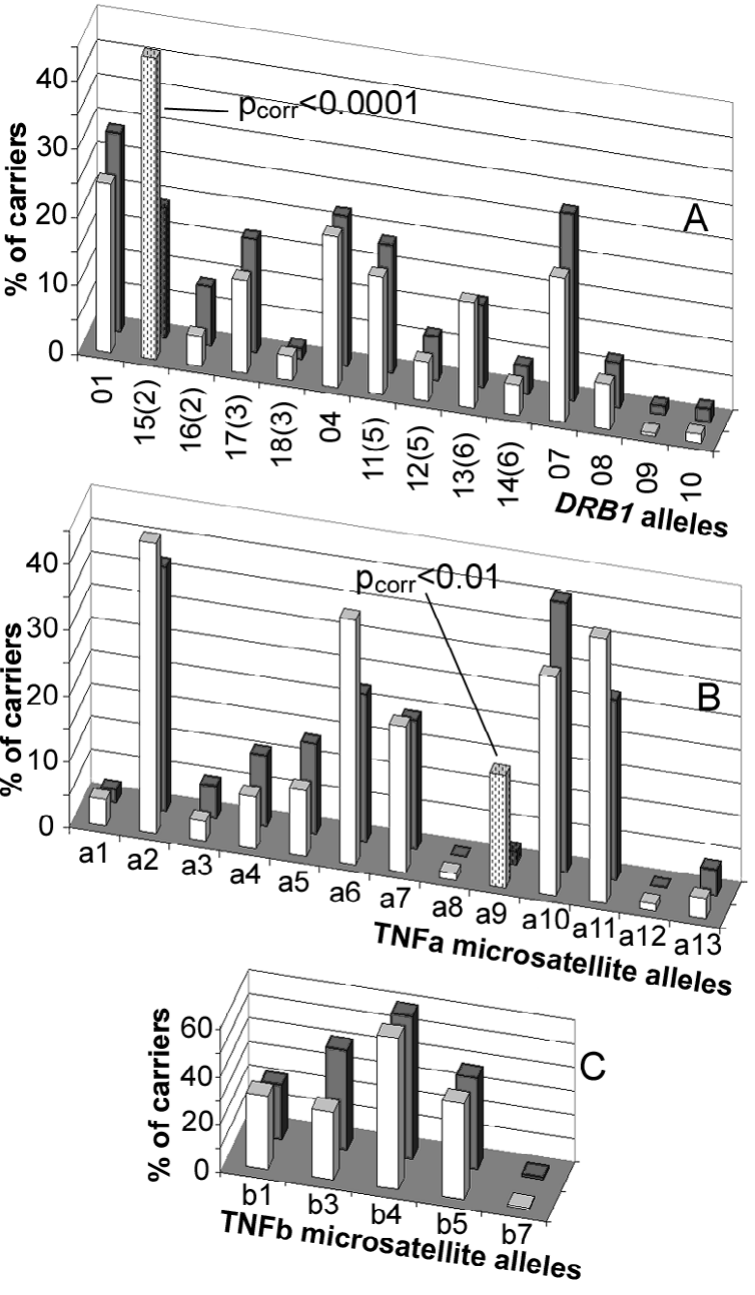
**Phenotypic frequencies (%) of some polymorphous HLA loci in the Russian population**. MS patients are shown by white bars and healthy individuals are shown by grey bars. The numbers of typed individuals are given in Table 1. **A: **HLA *DRB1*. **B: **TNFa microsatellite. **C: **TNFb microsatellite. For significant differences, *p*_*corr *_values are shown near the corresponding bars, which are shaded.

Individuals were genotyped at polymorphic loci at or near genes of the immune response situated at chromosomes 2, 3, 6 and 19 (Table 1). The allelic carriership (phenotypic frequency) in case of biallelic candidate loci (Figure 1) did not differ significantly in MS patients and controls. The allelic distributions of polymorphic candidate loci showed a positive association of MS with a carriership of allele group DRB1*15(2), corresponding to serological specificity DR15(2) (*p*_*corr*_<0.0001, OR = 3.1, CI is 2.1–4.6) (Figure 2A), and with a carriership of a TNFa*9 microsatellite allele (*p*_*corr*_<0.01, OR = 7.9, CI is 1.8–35.0) (Figure 2B). The allelic distribution of the TNFb microsatellite, which is adjacent to TNFa, did not differ significantly in MS patients and controls (Figure 2C). It agreed with TNFa/TNFb haplotype frequencies in patients and controls, which were estimated from genotype data via the population genetics data-analyzing software Arlequin version 2.00 [34]. The predisposing TNFa9 allele was part of three different haplotypes: TNFa9/TNFb1, TNFa9/TNFb4 and TNFa9/TNFb5 [see Additional File 1]. Both the HLA class II *DRB1 *gene and the class III TNFa microsatellite map to 6p21 and were in linkage disequilibrium; however, the DRB1*15(2)/TNFa9 haplotype frequency estimated from genotype data by the Arlequin software did not exceed 1% [see Additional File 2]. This suggested that DRB1 *15(2) and TNFa9 represented two independent MS-predisposing markers. The fact that no alleles of the tumor necrosis factor (*TNF*) and lymphotoxin α (*LT*) genes, also located at 6p21, differed significantly between MS patients and controls (see Figure 1A) correlated with the lack of strong linkage of *TNF/LT *haplotypes with DRB1*15(2) [see Additional File 3] and TNFa9 (not shown).

Application of the APSampler algorithm [20] identified five patterns that have a high probability of being associated with MS according to a multidimensional Wilcoxon-based criterion. All the patterns identified deal with carriership of alleles, without distinguishing homozygotes from heterozygotes. Two of the patterns were the single predisposing alleles DRB1*15(2) and TNFa9 discussed above, while the third pattern was a predisposing biallelic combination of *CCR5*Δ32 with DRB1*04, which was recently described for this data set [15,20]. Importantly, two new patterns comprising "trios" of allelic variants were also identified. The first pattern included the C allele of SNP -509 of the transforming growth factor β1 (*TGFβ1*) gene, DRB1*18(3), and the G allele of the cytotoxic T-lymphocyte antigen 4 (*CTLA4*) gene (trio 1). The second pattern included the alleles -238TNF*B1, -308TNF*A2 and again, CTLA4*G (trio 2). Fisher's exact test gave p < 0.01 for the association of MS with both patterns, ORs were equal to 18.0 (CI 1.0–330) for trio 1 and 17.4 (CI 1.1–300) for trio 2 (Table 2). To assess the probability that the trio patterns found were truly associated with MS, we also computed the false discovery rate, which was less than 0.003. The algorithm was intended to identify patterns as minimal allelic sets, in the sense that such a set provides stronger evidence of association with MS than any of its subsets. As shown above (Figures 1 and 2A), none of the alleles involved in the trios were an individual risk factor. For both trios, among individuals who have all the three alleles genotyped, the difference in phenotypic frequencies for two-element subsets between MS patients and healthy subjects was insignificant, with *p*-values always exceeding 0.025 (Table 2). So, both trios, which gain Fisher's test *p*-values less than 0.01, were correctly identified as minimal MS-associated allelic sets. The fact that two alleles of the *TNF *gene were required to form trio 2 was in agreement with the data suggesting that TNF*B1 and TNF*A2 are not parts of a single widespread haplotype [see Additional File 3].

**Table 2:** Phenotypic frequencies (carriership) of the three-allele combinations (trios) and their two-element subsets in Russian MS patients and healthy individuals

Allelic combinations	MS patients, N (%)	Controls, N (%)	*p-*value^#^	OR (95% CI)^##^
-509TGFβ1*C, DRB1*18(3), CTLA4*G (trio 1)*	5 (5.3)	0 (0)	**0.0086**	18.0 (1.0–330)
-509TGFβ1*C, DRB1*18(3)	5 (4.4)	2 (0.7)	0.029	
-509TGFβ1*C, CTLA4*G	63 (61)	102 (58)	0.61	
DRB1*18(3), CTLA4*G	5 (3.0)	2 (1.1)	0.26	

-238TNF*B1,-308TNF*A2, CTLA4*G (trio 2)**	11 (8.7)	0 (0)	**0.0033**	17.4 (1.1–300)
-238TNF*B1,-308TNF*A2	13 (9.9)	6 (5.4)	0.26	
-238TNF*B1, CTLA4*G	38 (30)	15 (17)	0.036	
-308TNF*A2, CTLA4*G	26 (16)	15 (14)	0.73	

* 99 MS patients and 154 controls were genotyped at these polymorphic loci of *TGFβ1*, *DRB1 *and *CTLA4 *genes.

** 126 MS patients and 87 controls were genotyped at these polymorphic loci of *TNF *and *CTLA4 *genes.

^#^*p-*values considered to be significant are given in bold face.

^## ^OR (95% CI) values are shown for significant *p*-values.

## Discussion

Based on the results presented, several (partially overlapping) subgroups may be identified in the common group of Russian MS patients, depending on carriership of distinct minimal patterns of susceptibility including one (DRB1*15(2) or TNFa9), two (*CCR5*Δ32, DRB1*04) and three (-509TGFβ1*C, DRB1*18(3), CTLA4*G or -238TNF*B1,-308TNF*A2, CTLA4*G) alleles of candidate genes. These results are evidence of the genetic heterogeneity of MS.

MS associations with DRB1*15(2) and TNFa9 were previously identified in our studies of independent groups of ethnic Russians [36,37] and were replicated here for a new dataset of patients and control subjects. Thus, the HLA class II DRB1*15(2) is validated in this study as being associated with MS in Russians as in most other Caucasians. As follows from our data, DRB1*15(2) and TNFa9 represent two independent predisposing markers, in agreement with the proposal that two MS susceptibility loci exist within the MHC [5,38].

In previous studies, identification of MS-predisposing combinations of allelic variants at multiple loci consisted in stratification of affected and unaffected individuals mostly according to the carriership of MS-predisposing DR2 haplotype or its constituents and a subsequent pair comparison of phenotypic frequencies of distinct alleles of another gene of interest in subgroups of MS patients and controls. This kind of analysis found reliable associations of MS in subgroups of DRB1*15(2) (or DQB1*0602)-positive or negative individuals with the carriership of some alleles of genes coding TCRβ receptor [14], TGFβ1 [16], CTLA4 [13], ICAM-1 [19] and interleukin 4 receptor [18]. In our recent studies, we have extended this approach and stratified individuals according to any *DRB1 *phenotypes. For DRB1*04-positive individuals, associations of MS with *CCR5Δ *32 mutation [15] and with alleles of myelin basic protein (*MBP*) gene [17] were found. The latter association was found also for DRB1*05 positive individuals [17].

A novel nonparametric methodology used in this paper provides the capability to explore potential combinations of more than two allelic variants of polymorphic candidate genes. Two previously unknown tri-allelic combinations: -509TGFβ1*C, DRB1*18(3), CTLA4*G and -238TNF*B1,-308TNF*A2, CTLA4*G, which perfectly separate cases from controls in the present sample, were identified. Importantly, at least within our data set, non-overlapping subgroups of individuals bearing predisposing trios 1 and 2 constitute about 5% and 9% of considered MS patients, respectively, and 0% of considered healthy controls (see Table 2). This suggests that carriership of these patterns might predict MS development. Due to the linkage disequilibrium of chromosomal loci, a genetic epidemiological approach cannot prove unambiguously that a disease-associated gene is causal. However, a biological role for the *DRB1*, *CCR5*, *TGFβ1*, *TNF *and *CTLA4 *gene products in the pathogenesis of MS is plausible, and supports the idea that the genes are actual MS susceptibility genes.

Trios 1 and 2 have striking similarities that may determine their MS-predisposing properties due to dysregulation of inflammatory pathways by protein products encoded by their genes. First, both trios include the allele G of the gene for co-stimulatory molecule CTLA4, which is an important inhibitor of T-cell activation [39]. Carriers of the *+*49CTLA4*G allele in exon 1, coding for the peptide leader sequence, are characterized by a reduced CTLA4 inhibitory function [40], i.e. by impaired negative regulation of the immune response. Second, both trios include alleles of cytokine genes which promote inflammatory immune response owing to decreased level of antiinflammatory cytokine TGFβ1 (trio 1) or increased level of proinflammatory cytokine TNF (trio 2). Indeed, it is known that the circulating concentration of TGFβ1 is under genetic control, being lower in carriers of the promoter *-*509TGFβ1*C allele, in comparison with *-*509TGFβ1*T allele [41]. The *-*308TNF*A2 allele included in trio 2 is more actively transcribed than -308TNF*A1 [42]. The sequence of the promoter region of -238TNF*B1, another trio 2 *TNF *allele, suggests that it is also associated with increased *TNF *expression [43]. In trio 1 (-509*TGFβ*1*C, DRB1*18(3), CTLA4*G) carriers, a two-hit repression of negative regulation of the immune response is coupled with a contribution of the HLA class II DRB1*18(3), which is not associated alone with MS (see Figure 2A). Characteristics of autoantigen presentation in MS by the products encoded by DRB1*18(3) have not been studied; however, it is clear that presence of this allele in trio 1 may be an important factor of the individual immune response regulation, supplementing effects provided by two other alleles of the trio.

Our results support the notion that genetic susceptibility to MS arises as a result of contribution of several predisposing alleles involved in the autoimmune inflammatory response. The nature of the interplay between the alleles in the trios remains unclear. If it arises as a result of a cumulative effect of multiple hits, these patterns provide a picture of small, individually insufficient changes combining to provide an overall significant response leading to predisposition to MS. However, this scenario does not exclude possible epistatic interactions of genes involved in the trios.

## Conclusion

The results presented here provide the first identification of a combination of more than two alleles conferring a genetic predisposition to MS as a complex polygenic disease. The two newly identified trios perfectly separate cases from controls, at least in the present sample, as would occur in a classic monogenic dominant disease, where all observed carriers are patients. Because of sample size limitations, the result can only be considered as preliminary. However, the biological properties of the genes included in the identified trios suggest a coherent picture of dysregulation of inflammatory pathways, implying the validity of the MS-associated patterns and encouraging efforts required to replicate associations in independent data. The APSampler algorithm used to identify the predisposing patterns is highly efficient, as it requires only hours of computation time on a laptop computer for this data set, and is flexible, as it can handle incomplete genotypic information. The algorithm provides a valuable resource for the growing volume of polygenic disease-related genomic data, allowing efficient exploration of such data to identify genetic predisposition and potential therapeutic targets.

## Competing interests

The author(s) declare that they have no competing interests.

## Authors' contributions

OOF and MFO set up the initial formulation of the problem. OOF organized the interaction between the medical, genetic and algorithmic groups. OOF, AVF, GP and MFO wrote the manuscript. AVF and MFO conceived of and developed the algorithm and the software. ANB and EIG diagnosed MS and recruited patients. ANB, MAS and TVA recruited healthy controls. ANB, EIG and OOF selected the candidate genes. TVA, MAS, ADA, and OGK performed genotyping analysis. OGK, MAS and OOF interpreted the patterns identified by the algorithm. MAS, TVA and AVF applied the software to the genotyping database developed by TVA. GP provided expert statistical advice used to significantly improve the algorithm.

## Pre-publication history

The pre-publication history for this paper can be accessed here:



## Supplementary Material

Additional File 1 **Additional Figure 1 – TNFa/TNFb haplotype frequencies in MS patients and controls **. Haplotypes are designated in accordance with TNFa/TNFb allele names. Click here for file

Additional File 2 **Additional Figure 2 – *DRB1*/TNFa haplotype frequencies in MS patients and controls **. **A: **DRB1*01 -DRB1*03 alleles; **B: **DRB1*04 -DRB1*09 alleles. Haplotypes are designated in accordance with *DRB1*/TNFa allele names. Haplotypes with frequencies less than 0.5% both in MS patients and in controls are not shown. Click here for file

Additional File 3 **Additional Figure 3 – *TNF/LT *haplotypes in MS patients and controls**. **A: ***TNF/LT *haplotype frequencies in all MS patients and controls; haplotype designations and entering SNP alleles are shown below. **B: **Frequencies of extended haplotypes including DRB1*15(2) and different *TNF/LT *haplotypes in all MS patients and controls. Haplotypes are marked in accordance with *DRB1 *allele name and *TNF/LT *haplotype designation given in **A**. For better visualization, the same axis scales were used. Click here for file

Additional File 4 Additional Table 1 – Phenotypic frequencies (%) of SNP alleles in Russian MS patients and healthy individuals Click here for file
