# Supplementary material for: Three allele combinations associated with Multiple Sclerosis
Source: BMC Med Genet. 2006 Jul 26;7:63. doi: 10.1186/1471-2350-7-63 (PMC1557481; doi:10.1186/1471-2350-7-63)
Supplement: Additional File 1 — Additional Figure 1 – TNFa/TNFb haplotype frequencies in MS patients and controls . Haplotypes are designated in accordance with TNFa/TNFb allele names. [file 1471-2350-7-63-S1.pdf]

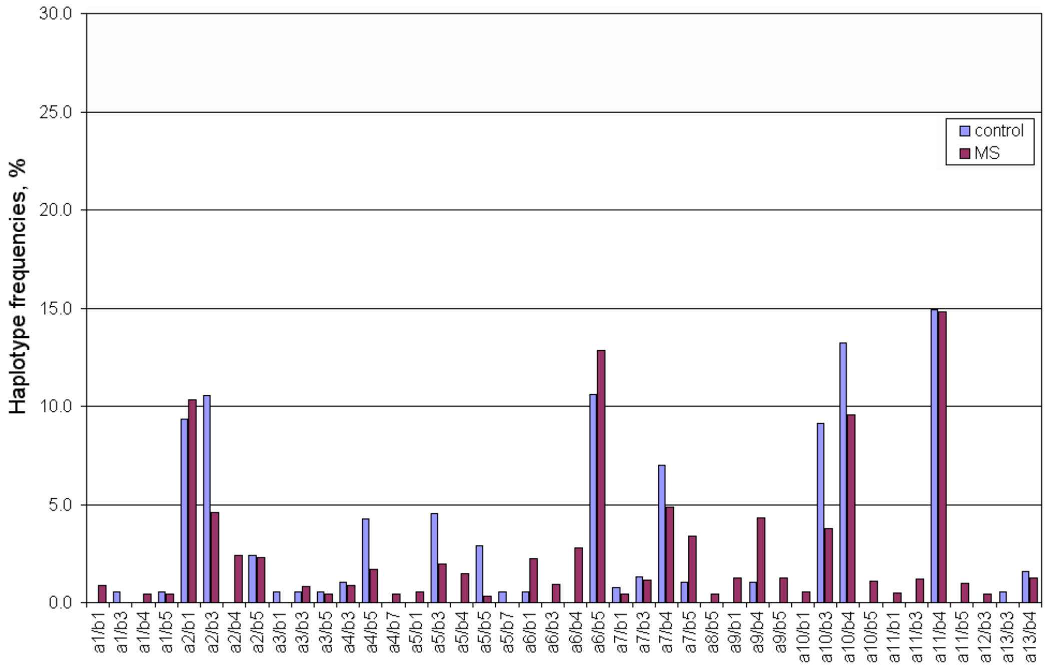

Additional Figure 1 *TNFa/TNFb* haplotype frequencies in MS patients and controls. Haplotypes are designated in accordance with *TNFa/TNFb* allele names.
