# Supplementary material for: Three allele combinations associated with Multiple Sclerosis
Source: BMC Med Genet. 2006 Jul 26;7:63. doi: 10.1186/1471-2350-7-63 (PMC1557481; doi:10.1186/1471-2350-7-63)
Supplement: Additional File 2 — Additional Figure 2 – DRB1/TNFa haplotype frequencies in MS patients and controls . A: DRB1*01 -DRB1*03 alleles; B: DRB1*04 -DRB1*09 alleles. Haplotypes are designated in accordance with DRB1/TNFa allele names. Haplotypes with frequencies less than 0.5% both in MS patients and in controls are not shown. [file 1471-2350-7-63-S2.pdf]

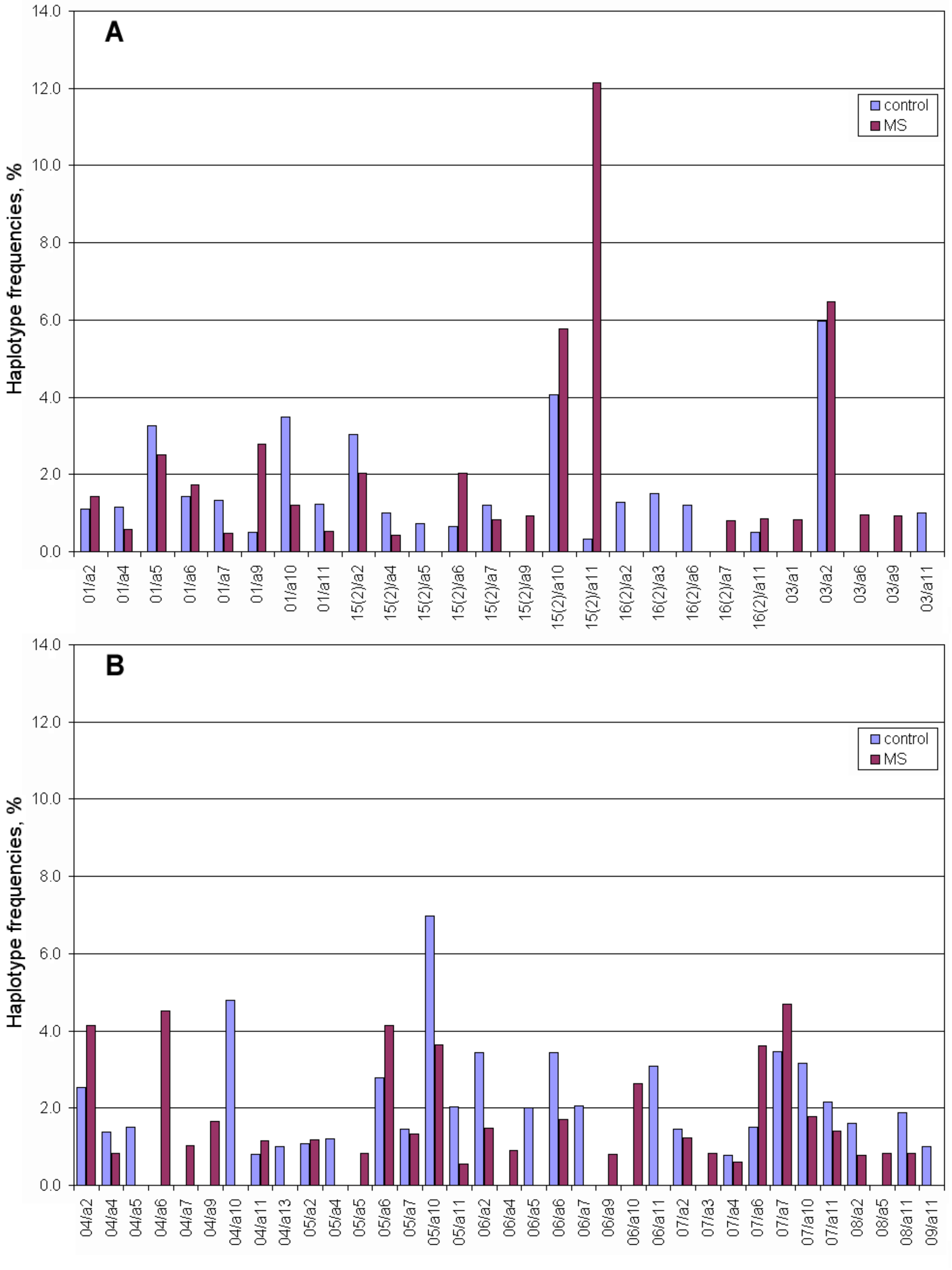

Additional Figure 2 *DRB1/TNFa* haplotype frequencies in MS patients and controls: (A) for *DRB1**01 -*DRB1**03 alleles, (B) for *DRB1**04 - *DRB1**09 alleles. Haplotypes are designated in accordance with *DRB1/TNFa* allele names. Haplotypes with frequencies less than 0.5% both in MS patients and in controls are not shown.
