# Supplementary material for: Three allele combinations associated with Multiple Sclerosis
Source: BMC Med Genet. 2006 Jul 26;7:63. doi: 10.1186/1471-2350-7-63 (PMC1557481; doi:10.1186/1471-2350-7-63)
Supplement: Additional File 3 — Additional Figure 3 – TNF/LT haplotypes in MS patients and controls. A: TNF/LT haplotype frequencies in all MS patients and controls; haplotype designations and entering SNP alleles are shown below. B: Frequencies of extended haplotypes including DRB1*15(2) and different TNF/LT haplotypes in all MS patients and controls. Haplotypes are marked in accordance with DRB1 allele name and TNF/LT haplotype designation given in A. For better visualization, the same axis scales were used. [file 1471-2350-7-63-S3.pdf]

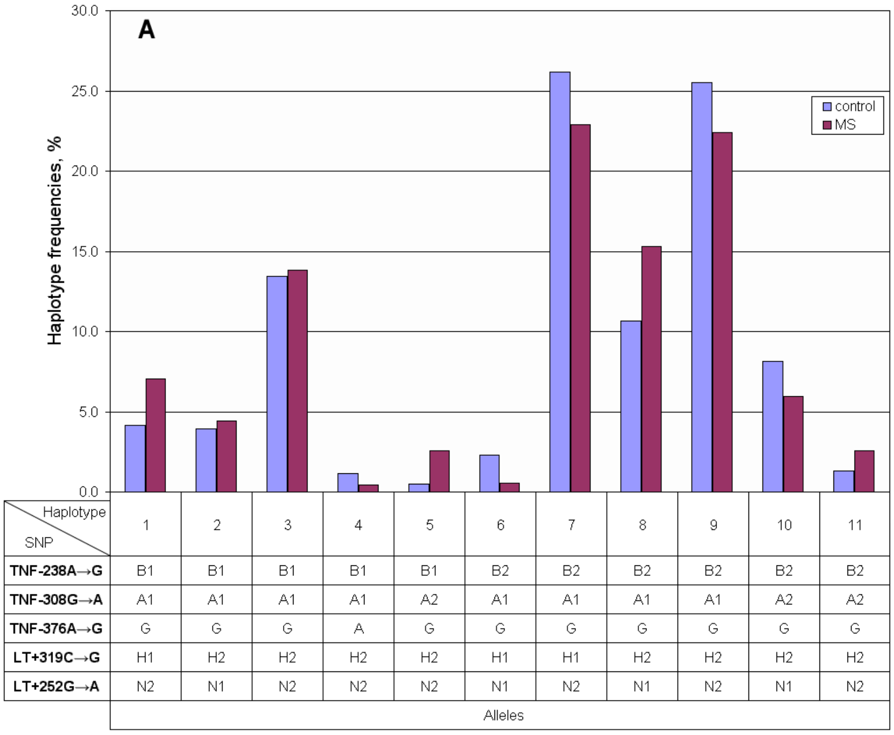

Haplotype / SNP	1	2	3	4	5	6	7	8	9	10	11
**TNF-238A→G**	B1	B1	B1	B1	B1	B2	B2	B2	B2	B2	B2
**TNF-308G→A**	A1	A1	A1	A1	A2	A1	A1	A1	A1	A2	A2
**TNF-376A→G**	G	G	G	A	G	G	G	G	G	G	G
**LT+319C→G**	H1	H2	H2	H2	H2	H1	H1	H2	H2	H2	H2
**LT+252G→A**	N2	N1	N2	N2	N2	N1	N2	N1	N2	N1	N2

Alleles

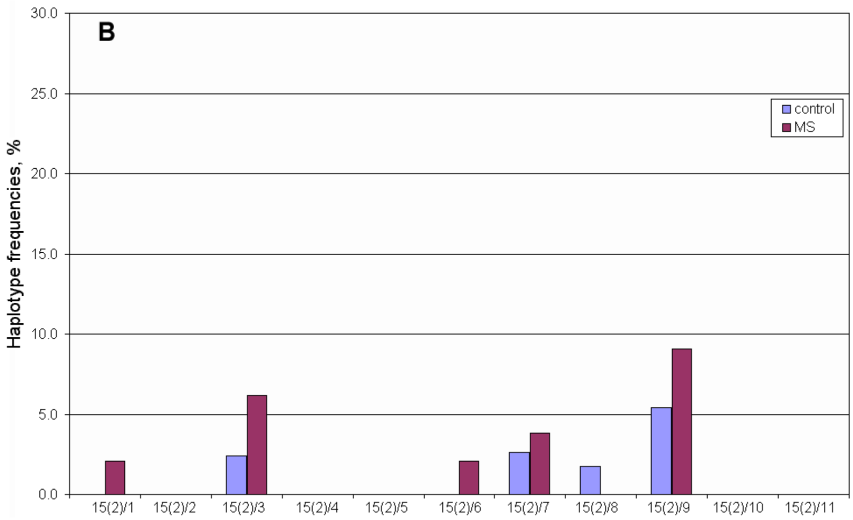

Additional Figure 3 *TNF/LT* haplotypes in MS patients and controls. (A) *TNF/LT* haplotype frequencies in all MS patients and controls; haplotype designations and entering SNP alleles are shown below. (B) Frequencies of extended haplotypes including *DR15(2)* and different *TNF/LT* haplotypes in all MS patients and controls. Haplotypes are marked in accordance with *DRB1* allele name and *TNF/LT* haplotype designation given in (A). For better visualization, the same axis scales were used.
