# Supplementary material for: Three allele combinations associated with Multiple Sclerosis
Source: BMC Med Genet. 2006 Jul 26;7:63. doi: 10.1186/1471-2350-7-63 (PMC1557481; doi:10.1186/1471-2350-7-63)
Supplement: Additional File 4 — Additional Table 1 – Phenotypic frequencies (%) of SNP alleles in Russian MS patients and healthy individuals [file 1471-2350-7-63-S4.pdf]

Additional Table 1 – Phenotypic frequencies (%) of SNP alleles in Russian MS patients and healthy individuals

SNPs	MS patients		Controls	
(Allele 1/Allele 2)	Allele 1	Allele 2	Allele 1	Allele 2
-308*TNF* (A2/A1)	20.3	100	21.7	100
-238*TNF* (B1/B2)	44.2	90.3	39.3	92.0
-376*TNF* (A/G)	3.0	100	3.4	100
+252*LT* (N1/N2)	46.1	95.6	42.0	92.0
+319*LT* (H1/H2)	57.2	92.0	59.2	91.2
-509*TGFβ1* (C/T)	86.6	55.5	84.4	62.0
+72*TGFβ1* (wt/ins)	97.0	16.2	98.5	12.4
+869*TGFβ1* (T/C)	84.0	60.0	82.7	64.9
+915*TGFβ1* (G/C)	97.5	14.0	97.6	13.3
+1632*TGFβ1* (C/T)	100	5.2	100	7.3
CCR5 (wt/Δ32)	98.2	24.2	99.4	19.2
+49*CTLA4* (A/G)	81.0	69.6	82.3	67.5

The numbers of typed individuals are given in Table 1.
